# An evaluation of the ethical- regulatory practices for genomic data sharing at three research-intense institutions in Uganda

**DOI:** 10.1186/s12982-026-03236-8

**Published:** 2026-11-09

**Authors:** Deborah Ekusai Sebatta, David Kyaddondo, David Kaawa-Mafigiri, William Buwembo, Shenuka Singh, Erisa Mwaka

**Affiliations:** 1 Department of Anatomy, College of Health Sciences, Makerere University, Kampala, Uganda; 2 Department of Child Health and Development Centre, College of Health Sciences, Makerere University, Kampala, Uganda; 3 Department of Social work and Social administration, College of Humanities and Social Sciences, Makerere University, Kampala, Uganda; 4 Discipline of Dentistry, University of KwaZulu Natal, Durban, South Africa

**Keywords:** Data sharing, Genomics, Regulatory documents, Document review

## Abstract

**Background:**

Despite the implementation of data protection regulations in several African countries, limited attention has been given to the governance of genomic data. This study used an evaluative research design to assess the ethical and regulatory practices for genomic data sharing at three research-intensive institutions in Uganda, with the aim of understanding capacity needs and identifying measures to strengthen responsible genomic data governance at the institutional level.

**Methods:**

Three institutions were purposively selected and anonymized as A, B, and C. A document review of eight genomic data sharing agreements (DSAs) was conducted, and key informant interviews with researchers, national research regulators, and lawyers were used as a form of validation. DSAs were requested because they are a requirement by the Uganda National Council for Science and Technology in collaborative research. A checklist was developed guided by the Malabo Convention and the Uganda Data Protection and Privacy Act. Data were analyzed using both deductive and inductive approaches. Five predefined domains guided the assessment: data ownership, protection and security, access, intellectual property, and benefit sharing. Emerging themes included informed consent, legal enforceability, and potential community harms.

**Results:**

The three institutions had DSAs; however, legal enforceability remains challenging. With the exception of one template, all agreements were signed and specific to genomic studies. Most DSAs stated that data ownership remained with the provider. Some of the agreements mentioned data protection, a few addressed intellectual property rights. None of the agreements included benefit-sharing statements, and the key informants confirmed the absence of a benefit-sharing framework. One institution had a legal office directly involved in research contracts, and some had functioning research ethics committees. Consent processes were largely regarded as procedural rather than meaningful, and concerns were raised about potential social harms.

**Conclusion:**

While all institutions had some basic guidance on data sharing, significant gaps remained, particularly in relation to benefit sharing, data protection, and legal oversight. The absence of statements on benefit sharing could be a fertile ground for exploitation. Institutions should strengthen consent processes and adopt participatory policy development approaches informed by frameworks like H3Africa. Legal and ethical support structures are also essential to safeguard the interests of emerging researchers.

**Supplementary Information:**

The online version contains supplementary material available at https://doi.org/10.1186/s12982-026-03236-8.

## Introduction

The rapid growth of genomic research presents exciting new possibilities in medicine, public health, and the life sciences [1]. With the ability to sequence entire genomes and analyze large sets of genetic data, we’re gaining deeper insights into human health, how diseases develop, and how treatments can be tailored to individuals ​. Worldwide, genomic researchers are increasingly dealing with vast data sets gathered by consortia [2]. Genomic research in Africa has also expanded significantly, largely driven by collaborative partnerships and at the heart of this is the sharing of associated participant data amongst researchers in Africa and across the world [3].

Of recent, many funders publishers, and other research institutions demand the sharing of research data because of its perceived benefits unless there are justifiable reasons [4]. Data sharing is the practice of making data available to other researchers by adding or combining research participants’ data into larger repositories [4]. The sharing of genomic data promises to increase research efficiency, expedite translation of research results, and ensure the traceability and transparency of published studies and maximize the utility of results [5–8]. In addition, by enabling researchers collaborate and work together, data sharing helps speed up scientific discovery and supports the translation of research findings into meaningful health interventions [9]. However, it raises questions on the ethical and regulatory arrangements for sharing the generated genomic data. Several countries in Africa have data protection regulations, however, there is no specific consideration for sharing genomic data [3]. With the increasing number of databases, however, has come a rising range of data sharing and privacy policies However, policy and practice are different.

While data sharing offers many benefits, the absence of clear regulation, well-defined DSAs, and supporting governance frameworks creates a risk of misuse. Instances of misuse of data sharing in genomic research have been noted with researchers in low- and middle-income countries (LMICs) affected more [10]. Examples of misuse include the alleged scandal involving the Wellcome Trust Sanger Institute in 2019 that proposed to commercialize a gene chip without proper legal agreements with partner institutions and the consent of thousands of African people. An increasing number of government departments, research communities, funding agencies, and scholarly journals are increasingly developing initiatives and policies to promote data sharing and greater access to data [11]. Specific to genomics, most funding agencies demand that sequence data be deposited in data repositories where other researchers can readily access it. However, researchers and institutions in low-resource settings are often constrained in their ability to use data not only because of limited capacity to analyze large datasets [12–14], but also due to absent or weak regulatory frameworks to govern responsible data use leading to inequity in genomic data sharing [14, 15]. There is a need for context-specific regulation of data sharing in genomics research in Africa. These advances also bring important ethical and regulatory questions especially around how genomic data should be shared.

The H3Africa initiative offers a model of participatory governance that emphasizes inclusivity, accountability, and shared decision-making in genomic research [16]. Despite its role in advancing data governance by establishing strict guidelines for collecting, storing, and sharing genomic data from African populations [17] regulatory gaps remain. In Uganda, various policies and laws have been formulated on data access, and protection. For example, the Data Protection and Privacy Act of 2019 which was established to protect the privacy and personal data of individuals by overseeing the collection and processing of personal information, thus safeguarding the privacy rights of those individuals whose data was collected. The challenge is that the Act does not offer adequate guidance on data generated from health research [18] as well as on genomics. An open data policy has been under development since 2017, whose goal is to make government-held data accessible to the public with minimal access restrictions. However, the policy is still in draft form and under review limiting enforceability. The National Council for Science and Technology (UNCST), responsible for research oversight in Uganda developed some guidelines on collaborative research data sharing [19], but are they were not very comprehensive. The revised 2025 UNCST Guidelines recognize participants’ interests in genomic data and require ownership arrangements to be negotiated and documented. However, by leaving ownership and benefit-sharing largely to contractual arrangements between collaborating parties, the guidelines provide flexibility but may also reinforce existing power imbalances. Although ownership is recognized normatively, effective control and decision-making authority over specimens and data may remain concentrated within institutions with greater resources and negotiating power. The guidelines do not establish sufficiently clear safeguards or oversight mechanisms to ensure that these arrangements are negotiated fairly and equitably or that the interests of participants and local institutions are adequately protected. This limitation reflects the approach taken in the 2014 guidelines, which stated that “each participating collaborative organization is left with the responsibility of safeguarding the rights and welfare of the research participants.” The statement means every organization involved in a collaborative research project is individually responsible for making sure that the rights, safety, and well-being of the respondents in the research are protected.

The aim of this paper was to examine the ethical and regulatory practices governing the sharing of genomic data in three research intense institutions in Uganda. This study assessed the ethical and regulatory practices for genomic data sharing in three research-intensive institutions in Uganda, with the aim of understanding capacity needs and identifying measures to strengthen responsible genomic data governance at the institutional level. The findings of this study will contribute to enhanced data governance and equitable data sharing in genomic research in low-resource settings. Understanding the regulatory practices governing data sharing in genomics research will contribute to enhancing institutional data governance in Uganda and similar resource settings.

## Methods

### Research design

An evaluative research design was utilized with multiple methods to examine the ethical and regulatory practices governing the sharing of genomic data in Uganda. In this study, ‘data sharing agreements’ (DSAs) refer to formal documents governing the sharing of genomic data, developed at the institutional level. The DSAs specify conditions for data access, use, storage and further sharing, and outline responsibilities related to data security, confidentiality, and compliance with ethical and regulatory requirements [23, 24]. In situations where standardized templates existed, these agreements were institute-wide and applied across genomic research projects, with limited project-specific variation, and were subject to review by institutional regulatory departments or legal offices. The DSAs were specifically requested from three institutions because they are a requirement by the UNCST which were used in collaborative research. The number of agreements reviewed reflects those that were made available to the research team. The institutions are delineated as A, B, and C to ensure anonymity. They were assessed for presence, adequacy and content to provide factual answers to the question about the regulatory practices guiding genomic data sharing and oversight mechanisms, their ability to address key ethical and legal requirements in genomic data governance. To strengthen the validity of the findings and support triangulation, we incorporated interviews with key informants. This work was part of a broader study which employed a systematic review as well as qualitative and quantitative methods, and some aspects of the work on data sharing have already been published [20–22].

### Sampling and setting

Purposive sampling was initially used to identify four established institutions conducting genomic research in Uganda. One institution declined to share the relevant documents and was therefore excluded, resulting in a final sample of three institutions. The three research-intensive institutions were selected based on their sustained publication records, extensive experience in international collaborations, and active engagement in genomic research in Uganda. The institutions selected represent a small sample of the limited number of institutions engaged in genomics research. The publication record was assessed through a review of publicly available peer-reviewed biomedical and genomic research publications produced by each institution over several years. They host several ongoing and completed genomics studies and maintain multiple collaborations with international partners. In addition, they employ researchers with hands-on experience in generating, managing, and sharing genomic data.

### Context

We define Research Ethics Committees (RECs) as independent institutional bodies responsible for reviewing and overseeing research involving human respondents in Uganda. In genomic research, they assess ethical issues related to consent, privacy, samples and data sharing, and help promote fairness and equity in research practices, in line with national guidelines, to protect the rights and interests of respondents and communities.

At the time of the study, DSAs were a relatively recent development within participating institutions. This evolving regulatory and institutional context influenced the number and types of DSAs that were available for review. In addition, in some cases, DSAs were not centrally stored, reflecting evolving institutional data governance practices. This context influenced the number and types of agreements that were available for review at the time of data collection.

Several observations were made during the process of obtaining administrative clearance to access the DSAs. When comparing the three institutions in terms of how they handled DSAs, we observed differences in points of engagement. At Institutions A and B, we interacted directly with the regulatory offices housed within the research departments. In contrast, at Institution C, all communication was routed through the legal office, making it the only institution where we interfaced with the legal team. We observed that not all institutions had RECs.

### Theoretical lens and application

This study was informed by two key theoretical frameworks: (1) the General Systems Theory (GST) by Von Bertalanffy [23], and (2) Rawls Distributive justice ​ theory . These theoretical frameworks guided data collection, analysis, interpretation, and reporting of findings. This study draws on the GST, which offers a useful lens for understanding the complex and interdependent relationships among actors involved in genomic data sharing. It is informed by principles originating from general systems theory, as articulated by Ludwig von Bertalanffy, particularly the emphasis on interdependence and feedback within complex systems.

In this study, GST was used to illuminate the interconnectedness within the governance system for genomic data sharing, examining how decisions and actions at one level shape, enable, or constrain other levels. By applying this perspective, we conceptualized genomic data sharing as a dynamic system rather than a series of discrete actions. This theoretical framework enabled analysis of genomic data sharing as a multi-level system, illustrating how structures, processes, and feedback loops shape ethical and governance outcomes. It also revealed that a failure at any single level for example, the absence of benefit-sharing provisions in DSAs can disrupt the entire system through feedback loops that erode participant trust and reduce willingness to share data. Building on this insight, while the GST was crucial in highlighting how gaps in institutional enforcement mechanisms represent component failures, it pays limited attention to power asymmetries embedded within collaborative research relationships. To address this limitation, the study integrates Rawls’ theory of distributive justice, which provides a normative lens for assessing fairness, equity, and the distribution of benefits and burdens in genomic data sharing. According to Rawls’, justice involves the fair allocation of resources, respect of the rights, and opportunities in a manner that respects each person’s equal claim to basic liberties. In this study, the main ethical issues that arise revolve around data ownership and sovereignty of genomic data, equitable access and benefit-sharing. To emphasize fairness, we invoke Rawls’ veil of ignorance principle a concept that challenges us to design systems without knowing our own position in society, encouraging decisions that are fair and impartial for everyone [21, 24]. Importantly, the study draws attention to how researchers and communities in LMICs often contribute valuable data but may be left out when it comes to accessing the shared data or its benefits [24], a concept that challenges us to design systems without knowing our own position in society, encouraging decisions that are fair and impartial for everyone.

### Tool development

Elements from the African Union Malabo Convention [25] and the Uganda Data Protection and Privacy Act [26] were used to develop the document review checklist. The Malabo Convention was selected because it provides a regionally relevant legal framework for data governance, privacy, cybersecurity, and cross-border data protection tailored to African contexts. The Uganda Data Protection and Privacy Act was included because it is the primary national legal instrument governing informed consent, privacy, and personal data protection in Uganda. The predefined domains were selected because they capture core legal and ethical obligations applicable to genomic data governance and mechanisms. Specifically, they reflect requirements related to custodianship and control of data, implementation of security safeguards, regulation of access and cross-border transfers, protection of intellectual contributions, and the need for equitable distribution of research benefits. The frameworks informed the development of a checklist comprising five evaluative domains: data ownership and sovereignty, data access, intellectual property rights, benefit sharing, and data protection and security. Domains relating to data ownership and sovereignty, data access, intellectual property, and benefit sharing were shaped by principles of distributive justice, reflecting concerns about fairness in collaborative research. Considerations around data protection, security, and the wider data-sharing environment were guided by the GST, which recognises the interconnected nature of technical, organisational, and governance arrangements. The checklist provided a structured basis for assessing the adequacy and ethical robustness of the data sharing agreements. Based on the study objectives, specific review questions were developed within each of these domains and systematically applied to the reviewed documents to guide coding and assess the comprehensiveness and ethical robustness of the data sharing materials. The documents were analysed to assess how well the data sharing agreements addressed these domains and to identify gaps that may affect institutional capacity for responsible genomic data governance.

### Legal expectation

Although the UNCST requires institutions engaged in collaborative research to use DSAs, the national law in Uganda remains largely silent on their application. In practice, institutions are expected to negotiate and formalize ​DSAs with their collaborators. The RECs typically request DSAs during the ethics approval process for studies that involve data sharing. However, responsibility for ensuring that these agreements are negotiated fairly, equitably, and in compliance with ethical and regulatory requirements rests primarily with the institutions themselves.

### Procedures

A structured checklist was used to guide the review process, ensuring consistency and alignment with the study objectives. Two reviewers independently assessed each document using the checklist, and any differences in interpretation were resolved through discussion and consensus. Although inter-rater reliability was not formally calculated, this process of independent review followed by structured discussions ensured consistency in coding and enhanced the trustworthiness of the findings.

For the qualitative arm, key informant interviews (KIIs) were conducted using a guide with stakeholders who were purposively selected because of their roles in the conduct, oversight, and regulation of genomic research. The analysis focused specifically on data from researchers, lawyers, REC members, and national regulators. These groups were selected because of their diverse and complementary roles in the genomic data sharing process. Researchers were selected because they are directly involved in obtaining informed consent as well as generating and sharing genomic data, lawyers provide legal guidance and oversight, REC members and national regulators are responsible for ethical review, governance, and enforcement of data sharing practices within research contexts. Data related to autonomy, consent, community considerations, and governance were drawn from the KIIs [20, 21]. Interviews were conducted in English through a combination of in-person and virtual formats, with the setting determined by the preferences and convenience of the respondents. Written informed consent was sought for all respondents. The interviews were conducted by the lead researcher (DES and a research assistant). The interview questions explored the ethical, social, and legal concerns related to sharing genomic data within collaborative research relationships. The interview guide comprised open-ended questions on stakeholder roles, consent processes, data-sharing practices, governance challenges, regulatory oversight, and perceptions of benefit sharing. The KIIs were reported in accordance with the Consolidated Criteria for Reporting Qualitative Research (COREQ) guidelines [27]. Relevant information on the study design, data collection, analysis, and researcher characteristics is provided in the methods section to enhance transparency and rigor.

### Analysis

We conducted directed (deductive) content analysis of the DSAs, guided by a structured checklist derived from existing literature to assess the presence or absence of key concepts on genomic data governance.

The analysis began with a close reading of each document to identify statements or provisions to identify the presence of specific statements or provisions highlighting all text that appeared to fall within the relevant categories [28] (Table 1). A coding framework was developed through a deductive approach [29], guided by existing literature [25, 26] and ethical guidance on genomic data sharing including the H3Africa Data and Biospecimen Access Policy and negotiating fairness [30, 31]. The Global Alliance for Genomics and Health (GA4GH) Framework ​ for Responsible Sharing of Genomic and Health. Guided by principles of distributive justice, interpretation of domains relating to data ownership, access, intellectual property, and benefit sharing focused on questions of fairness and equity in collaborative research arrangements. In contrast, considerations of data protection, security, and the broader data-sharing environment were informed by GST, which emphasises the interconnected nature of technical, organisational, and governance structures (Table 1).

Two researchers independently applied the coding framework using a predefined template and relevant text segments were highlighted and grouped according to the established categories. Each document was reviewed to determine whether the predefined concepts were explicitly addressed, and the findings were presented using frequency tables (Table 2). The descriptive frequencies were used only to map patterns across institutions. This process enabled us to clean the data and summarize the, which illustrated how often specific elements appeared across the reviewed documents. Consensus on the coding categories, sub-categories, and their definitions was reached through discussions with the broader research team [32]. Data from the document assessments, guided by the checklists were coded and entered into Microsoft Excel. Patterns across institutions were identified by examining how often predefined ethical and governance concepts were addressed in the documents. Narrative descriptions were then generated by synthesizing extracted text under each concept, allowing deeper examination of how these issues were addressed. They were used to explore how these concepts were articulated, emphasized, or omitted in practice. The analysis involved more than just checking for presence; we also examined how language was used to frame or convey meaning within each context. Narrative summaries were used to complement the frequency data, offering deeper insights into how key concepts were articulated or in some cases, omitted. Final consensus on coding categories, subcategories, and their definitions was reached through discussions with the broader research team.

**Table 1:** Coding framework for content analysis of key components in the review

Domains	Essential elements	Sub elements
1)Data ownership and sovereignity	Presence of the essential elements on data ownership	• Statement on data ownership • Statement on who owns the data • Statement on data shall not be used without the prior consent of the organization which shared the data • Requirements for sovereignty provided
2) Data protection and security	Statements on data protection and security	• Statement on data protection and security • Indication of what will be done in case of data breach
3) Data access and governance data sharing practices	Statements on data access	• Statements on how data will be accessed • Indication of platforms for data access • Statements on how to log in and upload data?
4) Intellectual property rights	Statements on intellectual property rights	• Statement on intellectual property • Statement on patent rights, royalties and terms and conditions
5) Benefit sharing	Statements on benefit sharing	• Mention benefit sharing • Statement on how benefits from the data will be shared

#### Analysis of the KII data

In addition to the deductive coding, an inductive approach was used to analyze qualitative data from the KIIs. Data analysis began with repeated reading of the interview transcripts to achieve familiarity with the data. The coding process was iterative: initial codes were compared across transcripts, refined, merged, or split as needed, and then reapplied to the data. A team-based approach was adopted for data analysis, involving DES and two social scientists, who developed a codebook [32]. Initial coding involved identifying recurring ideas and concepts within the transcripts. A preliminary codebook was developed and refined by three experienced researchers leading to the refinement of codes through iterative discussion rephrasing and the list of codes was cut down and merged [33]. Constant comparison across transcripts ensured consistency and analytic rigor. Finally, themes were developed by integrating inductive patterns with deductive categories, and these themes were reviewed, refined, and validated against the raw data to ensure coherence and representativeness.

## Results

A document review was conducted between August 4 and December 7, 2023. This review focused on DSAs from three selected institutions engaged in genomics research. A total of eight DSAs were reviewed across the three institutions. Four DSAs were obtained from Institution A, while two were obtained from each of Institutions B and C. This variation reflected the documents available and accessible at the time of the review rather than a predetermined sampling target for each institution. With the exception of one agreement at institution A, which was a template intended for use by all researchers involved in collaborative projects, most of them were fully completed, signed, and dated agreements for genomic studies between collaborating institutions.

The KIIs were conducted between August 2023 and March 2024 and involved 41 participants. This group included 15 researchers, 3 legal representatives, 14 REC members and 8 national regulators. Interviews revealed that, although Institution A has a DSA template in place, completed agreements are reviewed by members of the research leadership rather than by legal professionals. Although all three institutions specified platforms through which data could be accessed, only Institution A imposed strict confidentiality and non-disclosure obligations on data recipients. These provisions hold recipients accountable if the data are disclosed to an unauthorized third party, including through unintentional disclosure.

The team was informed that Institution C was the only institution where the legal team consistently reviewed DSAs. Review of the DSAs showed variation in the level of detail provided. While all agreements clearly outlined the roles and responsibilities of the parties involved, fewer of them provided contextual information such as the purpose of the study or the duration of the agreement. Information on the identities of the organizations was inconsistently documented, with some agreements providing clear institutional attribution and others offering more limited detail.

In some cases, documents displayed branding from more than one institution, indicating joint development or shared governance arrangements in data sharing processes. This reflects the collaborative nature of genomic research and the involvement of multiple actors in shaping data governance instruments.

A summary of the domains on data ownership and sovereignty, data protection and security, data access, intellectual property rights, and benefit sharing is provided across different institutions (See Table 2). The documents reviewed at Institution A provided the most detailed and explicit guidance, followed by those from Institution C, while Institution B’s documents contained the least detailed provisions. In particular, Institution A’s agreements voiced multiple governance elements, including data ownership, access procedures, data protection obligations, and oversight responsibilities, whereas Institution B’s documents addressed fewer of these elements and with less specificity.

Below is a summary of the DSAs across the selected institutions (Table 2).

1. Ownership and sovereignty

Data ownership and sovereignty refers to provisions specifying who has the decision making power, who holds authority over the data and statements on whether the originating institutions or countries retain control over data access, use, storage, and cross-border sharing. These elements were considered essential because they directly determine control, accountability, and protection of interests in the governance of shared genomic data.

**Table 2:** Presence of governance-related provisions in institutional genomic data sharing agreements across three institutions. Numbers presented (Table 2) indicate the count of provisions or statements addressing each governance element identified within the institutional genomic data sharing agreements. These counts reflect presence only and do not assess the depth, quality, or effectiveness of the provisions

Element	Institution A	Institution B	Institution C	Frequency
Data ownership and sovereignty	4	2	2	8
Data protection and security	2	0	2	4
Data access and governance of data sharing practices	3	0	2	5
Intellectual property	3	0	0	3
Benefit sharing	0	0	0	0

### Presence of the essential statements on data ownership and sovereignty

We reviewed the DSAs for the presence of essential statements on data ownership and sovereignty. The three institutions all had statements on who the data belonged to.

There was notable variation in how data ownership and use were articulated across the DSASs. While some agreements assigned exclusive data ownership to the provider, others allowed recipients limited rights to use the data through non-exclusive, purpose-restricted licences. The institutions demonstrated different approaches to data ownership specifications:

Both institutions A and B provided statements on who the owner of the data was. Below we selected one statement from one of the institutions.

*The provider shall be the sole owner of all the data and retains ownership of the data (Institution B)*

This means that the provider institution retains full ownership of the data and can decide how it is used and accessed. The recipient has no ownership rights and any use of the data is entirely dependent on permissions granted by the provider.

Institution C took a different approach by focusing on usage rights rather than ownership declarations. Instead of explicitly stating data ownership, Institution C granted recipients limited access through licensing arrangements, as exemplified by this statement:

*The data provider grants the recipient non-exclusive licence to use solely for use in the research programme (Institution C)*

A review of the DSAs revealed that Institution C had included an important additional clause stating that the data would be used exclusively for research purposes. This clause maintains data ownership with the provider but allows the recipient limited rights to use the data. The licence is non-exclusive, meaning the provider may permit others to use the same data, and the use is restricted to the specified research programme.

Although some agreements described local institutions as data providers and owners, one key informant noted that researchers in LMICs often lose control over their data once it is shared with collaborators from the Global North. This loss of control was attributed to the presence of stringent clauses embedded in research proposals and data sharing arrangements.

We do not have control once the data is sent out, especially to our people in the Global North. If data is sent there, sometimes we do not have control reason being, that we have limited resources, yes, maybe limited capacity to track, but also, the bargaining power. Most of the time, we are only receiving, and so, some of these proposals come in with stringent, should I say; attachments. So, you may find at the time of negotiation, you may not have the bargaining power to say how much you can protect the locals or protect yourself as an investigator (Regulator 01).

2. Data protection and security,

We define data protection and security as provisions specifying measures to safeguard data against unauthorized access, misuse, or disclosure, including requirements related to storage, access controls, confidentiality, and compliance with applicable data protection standards or regulations.

### Statements on data protection and security

Regarding data protection and security, only the DSAs from institutions A and C had statements on how data breaches would be handled. The DSAs varied in how data security and breach management were specified. Institution A outlined detailed and concrete data protection measures, including password-protected databases, restricted access to coded data, and physical storage of data in a locked study office. In contrast, Institution C referred more generally to the use of appropriate technical measures for data security and indicated that data breaches would be handled amicably, without specifying the procedures or safeguards to be applied. The agreements stated:

*Appropriate organizational measures should be taken against the unauthorized or unlawful processing of data or accidental loss or damage to the data. All data bases will be password protected. Study staff would receive only coded data. Data will be stored in a locked study office* (Institution A)

*Data breeches would be handled in an amicable manner*. *Appropriate technical measures to data security will be provided (*Institution C)

One key informant recommended legal protection as a deterrent to data misuse

To protect the participant’s data, the researcher should get into some sort of legal agreement with the people that they are sharing this data with. They might put caveats on how the data can be utilized and the extent it can be shared, that is if someone or an organization does contrary to what the agreement states, then they can be held accountable in that regard (R05).

3. Data access and governance of data sharing practices

For this review, data access refers to provisions specifying who is permitted to obtain and use the data, under what conditions, and through what mechanisms. We also searched for statements such as decision making structures and oversight mechanisms.

### Statements on data access and governance of data sharing practices

The DSAs varied in how they addressed data access, responsible use, and the governance of data-sharing practices. Only Institutions A and C included statements related to data access, but the level of detail and the nature of restrictions differed significantly.

Institution A took a very strict approach to data access that explicitly prohibited third parties from accessing the data.

“The Recipient shall not allow any third party, either directly or indirectly, access to the data” (Institution A)

This suggests a clearer governance structure in which access is tightly controlled and limited to the named partners, with no allowance for further sharing.

Institution C, by contrast, did not include explicit restrictions on third-party access. Instead, it described the platforms through which data could be accessed, including REDCap, *Microsoft teams and box platforms*.

However, the agreement did not specify who is permitted to use these platforms or under what conditions, indicating a gap in access governance and responsible use provisions.

Despite differences in how access and control were specified in the agreements, some researchers described data sharing as having expanded access to data for local investigators:

When I started working in research, it was mostly the collaborators who had access to data. So, I feel like things like the data-sharing arrangements have been a good thing for us, and they have opened up the space for investigators here to be able to use data that they are actively collecting (Researcher 08)

One REC member noted that with the risk of commercial value of data which was given free of charge, caution should be taken to limit access and commercialization risks

We have been receiving a request for the letters of support from institutions during client application for an unconditional permission for the unlimited access by third parties to data for genomic research and we were uncomfortable because we look at data as gold. It has a commercial value. The third parties that are going to have access to it, are going to have it, and apply it for commercial benefit and yet it was supplied by volunteers for no benefit, we thought that was unjust (REC 02).

4. Intellectual property (IP) rights: For the purposes of this review, IPs were understood as the provisions in DSAs that specified who may claim ownership or control over research outputs that arise from the use of shared data. These provisions typically address how discoveries, innovations, publications, or commercial products are attributed, protected, or shared among collaborating parties.

### Presence of statements on IP rights

Regarding IPs, only Institution A included explicit statements. These provisions addressed copyright and prohibited unauthorised copying or modification of the data.

*The data fully belongs to the provider institution and the copy rights prohibits unauthorized copying or modifications* (Institution A).

The statement means that the data is considered IP protected under copyright law. The recipient cannot copy, reproduce, alter, adapt, or modify the data without the provider’s explicit permission.

One legal professional noted that the practice in Institution C follows the university policy on IP.

*We follow the university policy on IP which states that if a researcher who is employed by the university discovers an IP it belongs to the university. In collaborative research*,* the IP either belongs to the university or there is co-ownership (Lawyer 02)*

Participants’ perspectives on IP revealed a tension between commitments to open data sharing for the public good and the need to protect IP, particularly where ownership and attribution were considered at risk:

One researcher alluded to the IP being owned by the researchers.

*The IP is owned by the PIs and we are open to sharing data but should we sense that if we share this data we can lose IP then our co-investigators may say no we are not open to sharing*,* we will put an embargo. We believe in immediate release for public good because the end of it*,* these are natural*,* God’s given. Just because you have sequenced shouldn’t give you exclusive rights in summary (Researcher 07).*

### Benefit sharing

In this review, benefit-sharing provisions refer to whether benefits arising from the use of shared data are addressed, when and with whom benefit-sharing discussions should occur, which stakeholders should participate in these discussions, and who is identified as a potential beneficiary.

### Presence of statements on benefit sharing

As shown in Table 2, none of the DSAs had statements on benefit sharing across the institutions.

The dearth of any reference to benefit sharing in the reviewed documents may reflect limited institutional guidance or the failure to incorporate existing guidance into DSAs, raising concerns about inequity and the potential exploitation of data providers and source communities.

The lack of guidance on benefit sharing was confirmed by a REC member.

Right now, in our guidelines or in our laws we do not have any framework for benefit sharing for genomic research and so there is a potential for exploitation of the research participants and their communities because of lack of benefit sharing platforms (REC 03).

#### KII data

After revising the codebook, a second cycle of coding was conducted to identify relationships among codes which were then grouped into broader categories. This process led to the identification of three additional domains that were not explicitly covered in the DSAs​ but were raised consistently by participants. These included: (1) informed consent, particularly concerns around the consent processes; participant autonomy, (2) legal enforceability, highlighting gaps between existing legal frameworks and their implementation; and (3) community harms, reflecting fears of stigma, discrimination, and reputational damage (Table 3).

**Table 3:** Additional domains that emerged from the KIIs

Domain	Key issues identified
Informed consent	Concerns over informed consent processes Calls for culturally appropriate consent processes Participant autonomy.
Community harms	Risks of stigma, discrimination, and reputational harm to communities were highlighted.
Legal enforceability	Disconnect between legal policies and their practical implementation. Concerns about enforcement, institutional mandates, and legal remedies.

#### Informed consent concerns

The GST was used to examine concerns surrounding informed consent for genomic data sharing, particularly those related to power dynamics within the broader research system. Participants reported that although formal consent procedures were in place, including the use of separate consent forms for genetic testing, a gap remained between procedural compliance and consent that was meaningful and culturally grounded.

Participants raised concerns about limited transparency in data-sharing processes and reported that communities were often excluded from decisions concerning the sharing and reuse of research data. They emphasized the need for consent processes that are culturally appropriate and informed by meaningful community engagement.

Respondents emphasized the need for consent processes that are culturally appropriate and informed by community input. They advocated for meaningful community engagement in decisions about how data and research findings are shared, and stressed that consent should go beyond a one-time signature. Instead, it should be an ongoing process built on dialogue, transparency, and shared values. Respondents recommended meaningful involvement of communities in the consenting process to ensure that research data is shared in a community-informed and culturally acceptable manner.

One Key informant highlighted the need for culturally acceptable consent which engages the affected communities

*It is not all about making the participants sign different consent forms but there is need for a more culturally appropriate and community informed consent processes which are rooted in dialogue* (REC member 05).

Concerns were also raised about the challenges in maintaining privacy and confidentiality of the shared data.

The findings revealed concerns about confidentiality and privacy in genomic data sharing, primarily arising from limited confidence in institutions’ ability to protect participants’ identities when data are shared beyond the original research setting.

One of the key challenges identified is attributed to the fact that genomic information is inherently identifiable. When genomic data are shared, they are rarely limited to raw sequences alone. Researchers often request additional metadata, such as country of residence, to give context to the data. While this makes the data more scientifically valuable, it also significantly increases the risk of re-identification and breaches of privacy and confidentiality. Respondents highlighted the need for strict, context-sensitive guidelines and regulatory controls around genomic data sharing, as inadequate oversight could inadvertently expose respondents to harm.

One participant noted that in the absence of strict guidelines on the sharing of genomic data, the respondents are at risk of breach of privacy which is their right.

*So*,* these are all facets we are not thinking about very strongly. Not that the legal frameworks are totally missing. Because you see*,* we recognize privacy right from the Constitution of the country. And that’s the supreme law of the country. Privacy is a right. And taking away people’s data is stamping with their privacy* (Lawyer 01).

#### Community harms

There were deep concerns about the potential harm genomic research could cause to individuals and their communities, particularly when the implications of data sharing are not fully explained to research participants.

One major concern was the risk of social stigma and family disruption resulting from genomic findings that contradict individual or community identity. When genomic data, especially with accompanying demographic or personal information, are shared across institutions, or globally, the possibility of re-identifiability increases which could lead to social stigma. Respondents expressed fear that scientific results could be used to challenge their place in the community, leading to shame, rejection, or even breakdowns in family relationships. In such cases, research participation was seen not only as harmful but as a source of regret and betrayal, especially when respondents feel they were not warned about such risks.

These harms are deeply rooted in cultural and social identity, highlighting that data sharing in genomic research may carry consequences that go far beyond individual privacy breaches. Respondents felt that if results prove something different from what is culturally accepted or believed, they could face accusations, loss of social belonging, or serious domestic consequences.

One participant highlighted how a breach of privacy may lead to social harm

*When you submit it*,* you will say that all this sequence is from Uganda*,* collected from the central region. That’s already good enough to know that this person most likely was a muganda. You try as much as possible to remove somebody’s name and put XYZ 1992 and so on. You know*,* you put numbers so that somebody doesn’t really link that sequence back to the person. But I can guarantee you that*,* all genomics data will always link back to the community* (R:08).

#### Legal enforceability

Aspects of legal enforceability were examined through a GST lens, with particular attention to disconnections between formal legal policies and their practical implementation, including concerns relating to enforcement mechanisms, institutional mandates, and the availability of legal remedies. A significant disconnect between existing policies and the actual practices of genomic data sharing, particularly in low-resource settings. While institutions may have general guidelines or ethical expectations in place, these often fall short of providing clear, enforceable frameworks for how data should be governed, accessed, or tracked once shared. One key issue that was highlighted was the absence of mechanisms to monitor or track data after it has been shared. Once data leaves the provider institution, there is often no way of knowing who is using it, for what purpose, or whether the terms of use are being followed. This absence of procedures and processes undermines both institutional accountability and participant protection.

This disconnect is further compounded by the limited access and control experienced by local researchers or ethics committees. Despite contributing to data generation, local institutions are often excluded from decision-making about how and where the data is shared, stored, or used reflecting broader power imbalances in global health research.

## Discussion

This study, conducted across three research-intensive institutions, revealed that all institutions had established data sharing agreements (DSAs), indicating a positive step toward strengthening responsible governance in genomic data sharing.

The findings are an indication that the presence of DSAs, legal support and RECs which were present in some of the institutions are critical elements in the regulatory practice guiding the sharing of genomic data. However, beyond just having these agreements, the presence and completeness of regulatory documents are especially important for ensuring data protection and proper data governance at the institutional level. The results demonstrated that although these documents are in place, enforcing them remains problematic without adequate systems to monitor and track how shared data is used. This reveals a continuing implementation gap where governance frameworks exist formally but are undermined in practice by insufficient institutional capacity to oversee compliance and control downstream data usage.

For genomic data, robust regulatory frameworks are essential for protecting individual privacy, promoting the ethical use of data, and ensuring compliance with national and international standards. The study’s analysis and interpretation were informed by distributive justice theory [24] and GST. Distributive justice theory emphasized equitable access to data for researchers in LMICs, fair benefit sharing, and fairness in data ownership and intellectual property. It also helped examine whose interests are prioritized within regulatory arrangements characterized by unequal power relations and whether existing practices promote equity in LMIC settings. GST positioned data-sharing regulation within the broader governance system, drawing attention to how coordination, oversight, enforcement, and institutional capacity influence the effectiveness of regulatory mechanisms in Uganda.

The GST offers a useful lens for examining the ethico-regulatory framework as a whole, highlighting how different components work together to promote fairness and equity in the sharing of genomic data [23] .

Documents from Institution A were the most detailed, clear with multiple governance elements, including data ownership, access procedures, data protection obligations, oversight responsibilities, which were then followed by those at Institution C, while those at Institution B were the least detailed.

The comprehensiveness of the DSAs from Institution A may be associated with continuous staff training, extensive experience in collaborative research, strong negotiating capacity, and the presence of a REC. At Institution C, the quality of the DSAs may reflect the direct involvement of the legal department in reviewing agreements, while the availability of these documents may be supported by the presence of a REC and continuous staff training.

. The findings of this study align with previous research conducted in South Africa which showed that RECs play a critical role in promoting ethical and lawful data sharing by reviewing and providing oversight for data sharing agreements [34]. Critical aspects such as data protection in some cases and benefit sharing in all cases were missing which could place the respondents at risk of data misuse. Without enforceable privacy safeguards in data-sharing agreements, participants may face risks of data misuse, stigma, social exclusion, and loss of autonomy. Our findings are consistent with evidence from Kenya, where stakeholders emphasized the need for open-data policies that protect participant privacy, prevent data misuse, and safeguard communities from potential harm [35] .

During the review, we were aware of external influences because all institutions operated collaboratively in some cases, documents displayed branding from more than one institution, reflecting the collaborative nature of genomic research and the involvement of multiple actors in shaping data governance instruments. However, the absence of certain components in these documents such as benefit sharing could be attributed to external influence. External influence was assessed at the document level using indicators such as through visual features of the DSAs, such as multiple institutional branding and partner-driven documentation, which were interpreted as indicators of external institutional involvement in shaping the agreements. In the absence of proper guidelines or a clear framework for genomic data sharing, such imbalances in representation and other missing components in the documentation could put researchers from LMICs at a disadvantage or at risk of exploitation. The external influence was measured against the elements of the different documents by evaluating how external factors align with or deviate from the principles, benchmarks, and requirements outlined in these frameworks. Our interest was in understanding whether certain institutions were more significantly affected by these influences. The analysis suggested that external influences were more visible in some agreements than others, particularly where documents reflected multiple institutional branding. However, given the small number of agreements reviewed, these observations were interpreted cautiously. The findings on external influence align a review Modlin, Sugarman [36] conducted in LMICs that indicated that the external influence could contribute to imbalances in decision-making and leadership, which in turn may lead to an unequal distribution of influence which may not necessarily align with the needs of the local institution. These power imbalances have made African research partners to oftentimes remain silent about worrying inequalities. While the studies shed an important light on the issue of power differentials in collaborative research because of the unleveled ground [13, 37], we don’t agree that all researchers and especially the more experienced ones should remain silent about the inequalities. We recognize that some emerging researchers from LMICs could be compromised because several early-career researchers tend to lack the necessary skills to effectively negotiate during the development of regulatory documents.

The findings show that consent must go beyond a one-time signature, particularly in contexts demonstrated by power imbalances. Power imbalances operate at multiple levels during the consenting process, affecting both the relationship between research participants and researchers as well as the LMIC researchers and collaborators from the Global North. Several respondents perceive research participation as a favor granted by researchers which they often link to quick access to healthcare making them feel a sense of gratitude or indebtedness. Similarly, LMIC researchers may feel constrained by the funding and technical dependencies, limiting their ability to influence consent language or processes shaped by external partners. These dynamics undermine the voluntariness of consent and raise distributive justice [24] concerns about the fair allocation of risks, burdens, and benefits, particularly when researchers from LMICs and vulnerable respondents have little influence over how their data are ultimately used or who benefits from them. The data aligns with the views of McCormack et al. who noted that the views of respondents should be sought regarding use of their data [38]. The lack of transparency not only undermines trust but also infringes upon the autonomy of individuals and communities. The findings of the study agree with views of Nembaware et al. who indicated that respondents should be adequately informed about use of their data and they developed a framework to guide the process [39]. When respondents are not adequately informed about how their data will be utilized in the future, they are unable to make fully informed decisions regarding their participation. Autonomy is compromised when individuals are left in the dark about the implications of their consent, leading to a sense of powerlessness and disconnection from the research process. The consenting process should be a transparent practice that genuinely engages participants, ensuring they fully understand how their data may be used in the future.

A review by Walakira et al. (2024) on Uganda’s regulatory framework governing data sharing highlighted the power differentials between local and foreign research partners. The review contends that Uganda’s data sharing policy tends to favour foreign partners, who often hold more power and control over the data. This imbalance can lead to inequities in decision-making and the distribution of benefits, further disadvantaging local institutions and communities. This causes challenges in how local stakeholders and researchers may access, share, and use data from research projects (https://abbrs-uganda.org/wp-content/uploads/2024/07/ABBRS-Data-Sharing-Policy-Analysis-End-of-Project-Report-26.10.-23.pdf) [18]. The UNCST 2014 guidelines state that “each participating collaborative organization is left with the responsibility of safeguarding the rights and welfare of the research participants” [19]. However, do collaborating institutions from LMICs bear the entire responsibility of protecting the rights of research respondents involved in collaborative research? Collaborating research institutions in LMICs do not have the capacity or resources needed to fully protect the welfare of respondents whose genomic data is shared. This challenge could be attributed to unequal power dynamics leading to imbalances in decision-making between collaborating institutions and the increased risk of data misuse [12, 18, 40].

We noted that the institutions play a critical role in ensuring development and strengthening of the regulatory frameworks. It is critical to understand the role played by the institutional culture, norms, policies, and environment play in protecting researchers [34, 41]. Researchers from LMICs require continuous legal support in reviewing their documents and training and mentorship [42, 43]. Some organizations do not guide the process of managing data and don’t provide best practices for data management [34, 44]. Each institution must create its procedures because a lack of them places the institutions with weak systems at risk of exploitation. A study conducted in South Africa on data sharing and data governance found that the absence of institutional- and national-level frameworks contributed to researchers’ reluctance to share their data [45].

Another study conducted in sub Saharan Africa showed that a lack of research systems was a hidden concern by researchers from LMICs regarding data sharing [46]. Our thesis in line with the GST is that strengthening the regulatory systems at the three institutions will help safeguard the interests of both researchers from LMICs and research participants.

Important statements on benefit sharing were missing in all DSAs. With data currently being looked at as gold with a commercial value, the lack of clear statements on how benefits should be distributed, along with the absence of a guiding framework for genomic data sharing, jeopardizes researchers from LMICs, leaving them vulnerable to external influences and potential exploitation. While the experiences of developing regulations for genomic data sharing in high-income countries can offer valuable guidance, such regulations should not be directly adopted without adaptation to the African context (Staunton & de Vries, 2020). Similar studies conducted across Africa support the findings on the need for a benefit-sharing framework to guide fair and equitable distribution of benefits arising from research involving human respondents [14, 47–49]. The absence of clear benefit-sharing provisions in DSAs reflects and reinforces existing power imbalances, leaving local researchers and participant communities vulnerable to exploitative data use without fair return or recognition.

Our findings suggest that genuine ethical regulatory practices for genomic data sharing in Uganda must go beyond the mere existence of policies and agreements to address deeper concerns of fairness, power, and protection.

Guided by distributive justice, such a framework should promote the equitable distribution of the benefits, risks, and control associated with data sharing, including decisions concerning access, ownership, and intellectual property. Particular attention should be given to protecting the interests of research participants and local researchers who may operate under conditions of vulnerability. The framework should also require context-sensitive, iterative, and community-engaged consent processes. Strong data-protection safeguards should be central to the framework because inadequate protections may expose participants and institutions to data misuse, privacy violations, and exploitation.

This should entail systems working together and emphasise how ethical principles, such as fairness and data protection, can be operationalised through coordinated oversight, clear institutional responsibilities, and mechanisms to monitor data use beyond initial approval.

Beyond requiring DSAs, an ethical regulatory framework should assess whether data are appropriately prepared for international sharing. This includes determining whether participants understood that their data could be transferred and reused across national borders, whether adequate protections remain in place after the data leave the country, and whether local researchers and institutions retain meaningful involvement in decisions concerning their use.

There is need for a strengthened ethico- regulatory framework guiding the sharing of genomic data along with comprehensive DSAs relevant in regulating how data is shared individually or by organizations and this is in agreement with studies by [50, 51]. DSAs clearly describe what individuals are agreeing to and what responsibilities data stewards will undertake [52]. Institutions can adopt a participatory approach in policy development by actively involving a diverse range of stakeholders. This inclusive process helps ensure that policies are not only ethically sound, but also contextually relevant and culturally appropriate making them more likely to be widely accepted.

### Strengths and limitations

Examining documents from multiple institutions enabled comparative analysis, identifying commonalities and differences in practices and policies. The study identified institutions that are involved in a significant number of genomics research projects. However, including one institution as a benchmark or reference point could have provided a clearer standard for comparison. The study utilized elements from different tools which was critical in minimising bias. There were limited records of DSAs available for review, likely since genomics research is still a relatively new field in Uganda. Although we only reviewed documents from genomics studies. Selectivity bias is a likely weakness of document review but the researchers recognized it and were aware that their backgrounds shape their interpretation and thus they were mindful of it. We didn’t give weighting but checked to see which areas were available and noted the presence but there was no ranking.

The authors propose several solutions that could be put into consideration to address the gaps which are listed below.

Drawing inspiration from H3Africa’s governance framework, institutions can adopt a participatory approach in policy development, engaging researchers, ethicists, and community representatives in the process.

In the absence of detailed national legislation on data sharing agreements, institutions should strengthen their contracts management function to provide greater oversight and scrutiny of data sharing agreements. This includes involving multiple actors in the contracts process, such as research governance offices, legal counsel, ethics representatives, and data management units, to ensure that agreements are reviewed from ethical, legal, and operational perspectives. DSAss should be treated as core governance instruments that clearly define data ownership, access, intellectual property, benefit sharing, and decision-making arrangements. To be effective, these agreements should be developed early in the research process, supported by institutional legal expertise to ensure they are fair, enforceable, and aligned with national laws and institutional policies, and reviewed through ethics approval processes. In addition, mechanisms for follow-up and accountability should be established to ensure that agreed terms are implemented in practice.

Moreover, institutions should prioritize the development of well-defined policies and guidelines that outline the procedures for data sharing. These policies should clearly delineate when, how, and with whom data can be shared, ensuring transparency and accessibility for all stakeholders involved. Institutions should invest in legal resources to assist researchers in developing agreements that protect their interests while adhering to ethical and legal standards.

### Future research

Further research is needed to strengthen institutional practices and address the governance gaps identified in this study. First, empirical studies examining how DSAss are implemented in practice would help clarify the extent to which agreed terms on ownership, access, intellectual property, and benefit sharing are followed after data are shared, particularly on global platforms. Such work would provide evidence on where breakdowns occur between contractual commitments and actual practice.

Secondly, there is need for policy-oriented research examining how national legal frameworks could better support data sharing agreements, including mechanisms for oversight and accountability. This could help bridge the current gap between regulatory guidance and enforceable practice. Together, these areas of future research could contribute to more consistent, fair, and accountable data governance in collaborative research.

## Conclusion

Overall, all institutions had DSAss which represents a positive step toward promoting responsible governance in genomic data sharing. The findings underscore that the presence and alignment of multiple regulatory elements are essential for ensuring effective data protection and governance at institutional level. Some of the critical aspects like benefit sharing and data protection appeared to be missing in some documents. Drawing inspiration from H3Africa’s governance framework, institutions can adopt a participatory approach in policy development, engaging researchers, ethicists, and community representatives in the process. Additionally, providing legal support is essential, particularly for emerging researchers who may be unfamiliar with the complexities of data sharing agreements. The importance of benefit sharing cannot be overstated in the context of genomic research. Furthermore, institutions should consider the implications of data protection in their data sharing agreements.

### Implications for practice

Institutions should ensure interconnectedness among all components that guide the sharing of genomic data. All DSAs should align to a common minimum standard to ensure consistency in how genomic data is shared and protected. Institutions without established RECs should prioritize setting them up. Additionally, all institutions should ensure they have access to appropriate legal support to guide the development and enforcement of data and the statements in the documents.

## Supplementary Information

Below is the link to the electronic supplementary material.

Supplementary Material 1.

## Data Availability

All the results and details of the analysis have been presented in this manuscript. The tools have been shared as supplementary items.

## Abbreviations

DSA: Data sharing agreements
EU: European Union
IP: Intellectual property
LMICs: Low- and middle-income countries
MTAs: Material transfer agreement
RECS: Research ethics committees
SOP: Standard operating procedure
UNCST: Uganda National Council for Science and Technology
