# Supplementary material for: An evaluation of the ethical- regulatory practices for genomic data sharing at three research-intense institutions in Uganda
Source: Discov Public Health. 2026 Nov 9;23(1):1776. doi: 10.1186/s12982-026-03236-8 (PMC13748262; doi:10.1186/s12982-026-03236-8)
Supplement: Supplementary file 1 [file 12982_2026_3236_MOESM1_ESM.pdf]

Interview guide for key informants at Makerere University and affiliated research institutions

Introduction: Thank you for accepting to take part in this study looking at the perceptions of regulators about data sharing. The findings from this study will contribute to the development of guidelines that will provide protection for researchers and research participants involved in collaborative research

We are going to talk about data sharing in genomic research

A. Data sharing in genomic research

1. Can you kindly share your experience with collaborative genomics studies which with a data sharing component?
2. Can you kindly share the benefits of data sharing?
   Probe for efficiency, data science

B. Ethics of sharing

1. Can you kindly share the ethical and legal implications of data sharing in genomic research?
2. Probe for: discrimination, stigma, data access, data ownership, intellectual property rights

3. What are some of the ethical issues around data sharing in genomic research?
   Probe for data breaches, breach of confidentiality, re-identifiability

C. Benefit sharing

1. What are some of the different benefits that likely to accrue from shared genomic data?
2. What are the challenges around benefit sharing from genomic data?
3. In your opinion, what are the different types of benefit sharing that should be considered during sharing of genomic data?
4. In your view, who should benefit sharing be discussed with? (probe for research participants, researchers, funders)
5. What recommendations can you make regarding benefit sharing from shared genomic data?

Do you have any further questions or comments on what we have been talking about?

Thank you very much for your time and comments
